# Park the loop: An effective microsurgical tying technique

**DOI:** 10.1016/j.jpra.2025.01.009

**Published:** 2025-01-28

**Authors:** Florin-Vlad Hodea, Andreea Grosu-Bularda, Cristian-Sorin Hariga

**Affiliations:** a University of Medicine and Pharmacy Carol Davila, Bucharest, Romania; b Clinic of Plastic and Reconstructive Microsurgery, Clinical Emergency Hospital Bucharest, Bucharest, Romania

**Keywords:** Microsurgery, Technique, Knot, Tying, Suture

## Introduction

Microsurgery represents a set of high precision techniques that require appropriate training to improve skills and functional results. Microsurgical knot-tying is a critical skill in microsurgery, requiring precision and efficiency to ensure successful outcomes. Various techniques have been developed to enhance the efficiency and reliability of knot-tying in microsurgical procedures. Traditional microsurgical knot-tying techniques often involve inefficient maneuvers that can be challenging in most situations, especially for novice surgeons. These techniques may also lead to issues such as the short end of the suture adhering to surrounding tissues, making it difficult to re-grasp and complete the knot, ultimately losing time and gradually amplifying intraoperative fatigue.^1^

Some techniques were already described, each with different difficulty. The 'Airborne' Suture Tying Technique for Microvascular Anastomosis represents one such technique in surgical practices aimed at enhancing the efficiency and effectiveness of microsurgical repair. This method focuses on keeping the suture end elevated above the surgical field. By maintaining the short suture in an airborne position, microsurgeons can significantly reduce the time required to complete the anastomosis procedure, achieving time savings of approximately 15 to 20 percent.^1^ The 'through-the-loop' technique was introduced as a viable alternative method with easier manipulation of the short suture through a formed loop bent by the opposing instrument.^2^^,^^3^ Another technique that prevents the short end from touching the surrounding tissues is called” the chopstick rest technique”, wherein the short end of the suture is placed over the microforceps, which may render the process slightly more difficult.^4^

### Operative technique

Building on the principles of always controlling the short end of the suture, the ‘Park the Loop’ technique is a new method we designed to further enhance the efficiency and reliability of microsurgical knot-tying. It involves creating a loop in the long end of the suture and temporarily parking this loop within the curve of the short end, held slightly bent by either of the needle holder or micro-forceps, allowing for easier manipulation and knot completion.

**Figure 1:**
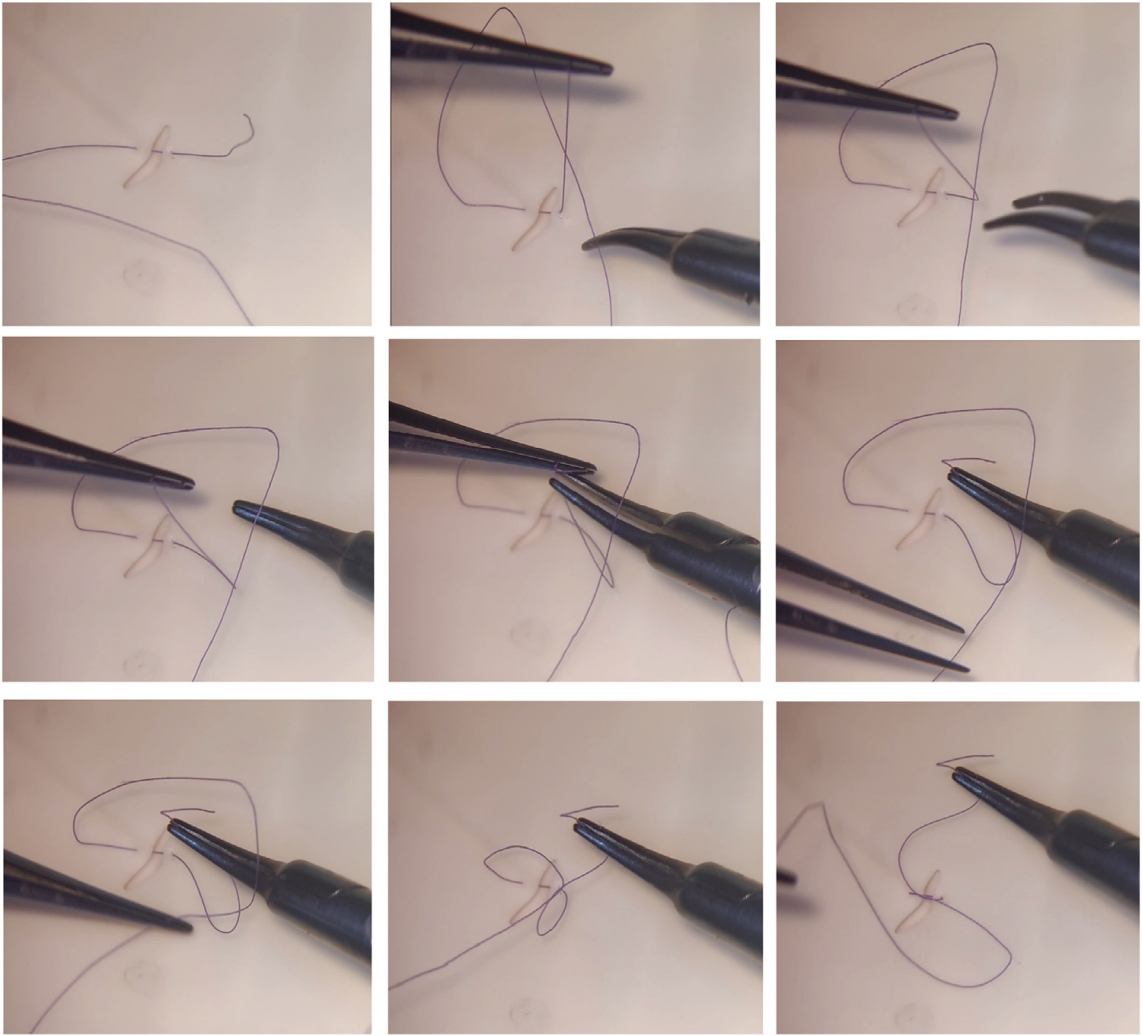
Suturing simulation on latex model. Top row: left - suture passed through both lumens, center: long end grabbed to form a loop, right - loop parked within the curve of the short end and long end released; Middle row: left – needle holder passed under the parked loop, center: needle holder grabs the short end, right – micro-forceps lets go of short end and moved towards long end; Bottom row: left – micro-forceps grabbing long end, center: suture ends are pulled in opposite directions, right – final aspect of finished throw.

**Figure 2:**
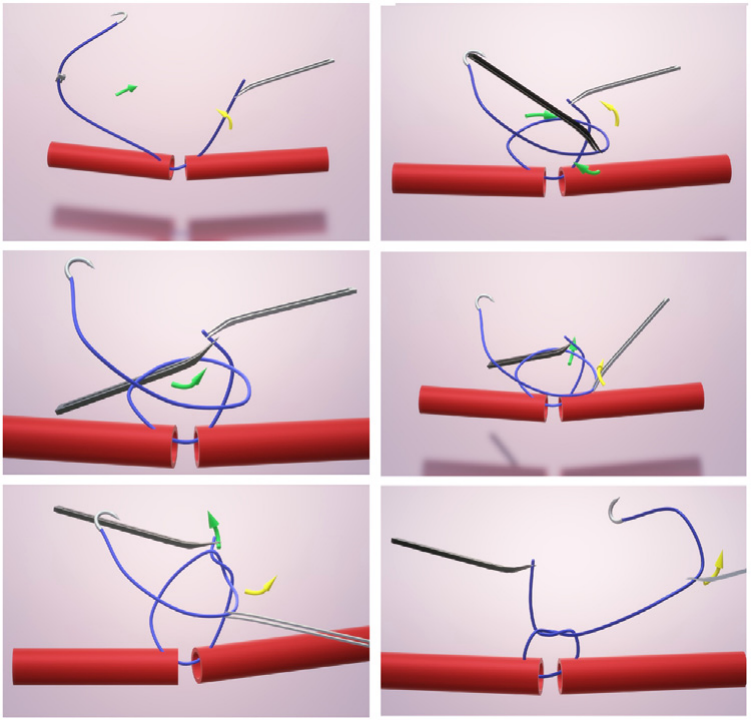
Schematic representation of suturing sequence. Top row: left - suture passed through both lumens, right - long end parked within the curve of the short end; Middle row: left - change of forceps position to grab the short end from under the parked loop, right - change of forceps position from short end to grab long end; Bottom row: left - creation of loop by pulling up the suture ends oppositely, right – final aspect of finished throw.

The ‘Park the Loop’ technique can be divided into three main steps as seen in Figure 1, Figure 2 or by accessing supplementary Video 1 on silicon tube model and Video 2 on latex model. First, slightly grasping the short end and bending it towards anastomotic site, so that the short end will be in a sagittal axis. Secondly, forming the loop in the long end of the suture perpendicular to the short end in a transverse axis, parking the long end loop and letting it go. The long suture will stay in place due to the spring tension, which will push the free end towards the short end, locking it into position. Thirdly, the instrument change and grabbing the short end from under the long loop. The first instrument that held the short end is let go and moved onto the long end to complete the throw. This is repeated for the next throws.

## Discussion

The ‘Park the Loop’ technique has the potential to improve the efficiency and success rate of microsurgical procedures by minimizing the risk of suture adherence and simplifying the knot-tying process. Having more efficient movement during anastomoses, surgeon fatigue is reduced while enhancing control of the microsurgical site. A particular benefit to this technique is when the short end is left longer, which can facilitate parking, useful in backwall first anastomoses. It may aid in settings where freestyle knot tying based on the surgeon preference and the need for adaptability in each situation. This adaptability allows for a more tailored approach to each unique surgical scenario, ultimately improving patient outcomes and satisfaction. One advantage of this technique is the permanent grasp on the short end of the suture. Surgeons can quickly acquire the necessary skills to implement this method, making it accessible across a wide range of microsurgical settings. Furthermore, the Park the Loop technique is compatible with both curved and straight instruments, enhancing its applicability. In conclusion, we consider this technique valuable and applicable in different microsurgical scenarios.

## Funding

None.

## Ethical approval

Not required.

## Conflicts of interest

None declared.

## Appendix Supplementary materials

Video 1. Silicon tube model knot tying simulation. Image, video 1

Video 2. Latex glove model knot tying simulation. Image, video 2
